# Microbial Fuel Cell-Based Biosensors

**DOI:** 10.3390/bios9030092

**Published:** 2019-07-23

**Authors:** Yang Cui, Bin Lai, Xinhua Tang

**Affiliations:** 1 School of Civil Engineering and Architecture, Wuhan University of Technology, Wuhan 430070, China; 2 Systems Biotechnology Group, Department of Solar Materials, Helmholtz Centre for Environmental Research—UFZ, 04318 Leipzig, Germany

**Keywords:** microbial fuel cells, biosensors, environmental monitoring, BOD, toxicity

## Abstract

The microbial fuel cell (MFC) is a promising environmental biotechnology that has been proposed mainly for power production and wastewater treatment. Though small power output constrains its application for directly operating most electrical devices, great progress in its chemical, electrochemical, and microbiological aspects has expanded the applications of MFCs into other areas such as the generation of chemicals (e.g., formate or methane), bioremediation of contaminated soils, water desalination, and biosensors. In recent decades, MFC-based biosensors have drawn increasing attention because of their simplicity and sustainability, with applications ranging from the monitoring of water quality (e.g., biochemical oxygen demand (BOD), toxicants) to the detection of air quality (e.g., carbon monoxide, formaldehyde). In this review, we summarize the status quo of MFC-based biosensors, putting emphasis on BOD and toxicity detection. Furthermore, this review covers other applications of MFC-based biosensors, such as DO and microbial activity. Further, challenges and prospects of MFC-based biosensors are briefly discussed.

## 1. Introduction

**Figure 1:**
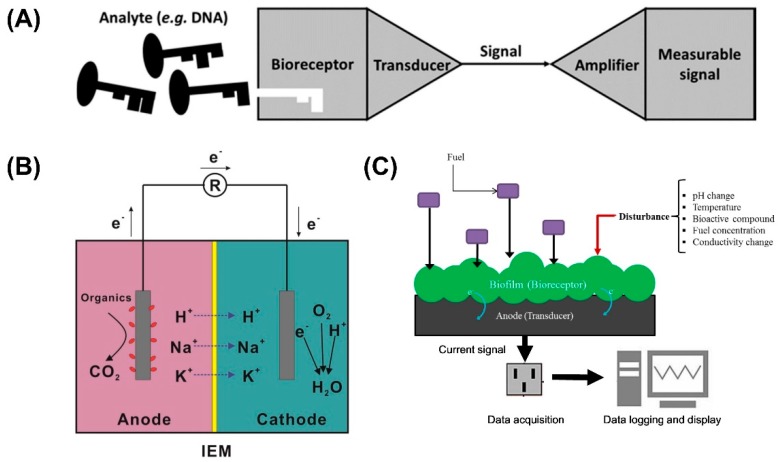
Schematic diagrams of (**A**) a biosensor [20], (**B**) a dual-chamber microbial fuel cell (MFC) and (**C**) an MFC-based biosensor.

Sensors can detect the properties and events occurring around and convert the sensed information into signals [1]. Nowadays, they are of great significance in many industries, providing quantitative information for counting, sorting, reading, and robotic guidance [2]. For environmental monitoring, stricter regulations and higher standards have resulted in growing demand for sensors that can detect pollutants quickly and sensitively. Due to the feasibility of portability, of working on-site and determining biological effects, biosensors have been emerging as appropriate analytical tools for environmental monitoring [3]. Typically, biosensors (Figure 1A) consist of biological recognition elements and physical transducers translating the biologic response into an electrical, thermal, or optical signal correlated to the analyte concentration [4]. According to the type of biorecognition, biosensors can be categorized as immunosensors [5], enzymatic biosensors [6], DNA biosensors [7], cell-based biosensors [8], and biomimetic biosensors [9]. Enzymatic biosensors occupy a considerable proportion in biosensors, and they exhibit high selectivity in differentiating the target [10]. However, they have several main limitations, such as cost-intensive approaches for enzyme purification and immobilization and short half-life time of enzymes compared to chemical catalysts [11]. These can be overcome by whole-cell based biosensors, and thus they are often considered as future alternatives to enzymatic biosensors [12,13].

As whole-cell based biosensors, microbial fuel cells (MFCs) have been applied for environmental monitoring. MFCs are devices that use microorganisms as catalyst to convert chemical energy directly into electricity [14]. According to the configuration, they can be classified into two types: single-chamber MFCs and dual-chamber MFCs. Dual-chamber MFCs (Figure 1B) consist of anodic and cathodic chambers separated by an ion exchange membrane (IEM), while single-chamber MFCs are mainly composed of anodic chambers and air cathodes [15]. Electroactive microbes are inoculated into the anodic chamber, where they generate electrons and protons by oxidizing organic compounds. Electrons are captured by the anode and then arrive at the cathode through an external circuit. Meanwhile, protons and other cations (e.g., Na^+^, K^+^) migrate to the cathode through the IEM to keep charge balance [16]. Finally, when oxygen acts as the electron acceptor, electrons and protons will combine with oxygen to form water [17]. Despite numerous improvements have been made in MFCs, low power output still constrains their utilization as high-power generators in practice. MFC-based biosensors inherit the common problems of MFCs, such as stability, reproducibility of the signal, long-term operation and substrate-induced metabolic cross-effects. However, MFC-based biosensors focus on the (linear) relationship between the signal output (e.g., voltage, current) and changes in environmental conditions other than high power output [18]. In an MFC-based biosensor (Figure 1C), the developed biofilm in the anodic chamber plays the role of the bioreceptor, while the anode is regarded as the transducer. The response of the anodic biofilm to the disturbance affects the electron flow rate, which is transduced into a measurable signal [19]. MFC-based biosensor is expected to be one of the most promising applications of MFC-derived technologies, which has been studied to measure various parameters, including biochemical oxygen demand (BOD), chemical oxygen demand (COD), volatile fatty acids (VFAs), dissolved oxygen (DO), toxicants, and microbial activity (Table 1).

In this review, we summarize the status quo of MFC-based biosensors, focusing on BOD and toxicity detection. Other applications of MFC-based biosensors, such as DO and microbial activity, are briefly addressed. Finally, the further challenges and prospects of MFC-based biosensors are discussed.

## 2. MFC-Based Biosensors for BOD Detection

BOD, one of the most widely used parameters for water quality assessment, is a comprehensive indicator of biodegradable organic compounds in water. Normally, a five-day BOD test (BOD_5_) is used to determine the standard BOD (unit of mg/L), which is evaluated by the amount of oxygen consumed through the respiratory activity of microorganisms in the sample after incubation at 20 °C for 5 days [44]. Since this method is time-consuming and labor-intensive, developing an alternative method that is fast and convenient to monitor BOD on site is necessary. Karube et al. [45] for the first time proposed the use of MFCs as a BOD sensor. *Clostridium butyricum* bacteria was immobilized on the electrode in the anodic chamber and a linear relationship between current output from the MFC and the BOD (glucose–glutamic solution standard) concentration were observed, which confirmed the feasibility of MFC-based BOD sensors. After that, several kinds of MFC-based BOD sensors were reported, and various kinds of microorganisms were employed [46,47]. MFCs with electron-mediators were also studied as BOD sensors [48,49], where the mediators were used to facilitate electron transfer from the microbial cells to the electrode, but these sensors suffered from the instability over long-term operation because of the toxicity of mediators to microorganisms. Accordingly, Chang et al. [23] proved that a mediator-less MFC could be used to continuously measure the BOD of wastewater for real-time monitoring. Moreover, an MFC-based biosensor was reported to be operated over 5 years in a stable manner [50], which was much longer than that of previously reported BOD biosensors (7 to 140 d) [51]. This demonstrated the advantage of MFC-based biosensors during long-term operation. Compared with conventional biosensors, MFC-based biosensors directly use the measured current or voltage as the output signals [52], so they can be processed and displayed more conveniently. Furthermore, they can also be designed and applied for remote areas due to their capability of self-generation of electric powers [53].

### 2.1. Environmental Parameters

There are many factors that can affect the performance of MFC-based biosensors for BOD monitoring, such as temperature, electrolyte conductivity and pH [54], feeding rates and water compositions [23,55]. Peixoto et al. [54] evaluated the effect of environmental changes on the performance of the biosensor using domestic wastewater with a BOD_5_ of 144 mg/L. The current density output increased by 6 mA/m^2^ while the temperature raised by 1 °C within the range between 11 °C and 33 °C. Similar effect was observed in this study for the conductivity of electrolyte (i.e., domestic wastewater) as well: altering the conductivity from 1.1 to 7.51 mS/cm improved the current output from 199 to 316 mA/m^2^. Moreover, maximum current density (288 mA/m^2^) was observed at the pH of 7.0, while either acidic or alkaline condition would decrease the current density (186 mA/m^2^ at the pH of 6.0 and 184 mA/m^2^ at the pH of 8.0). Chang et al. [23] addressed the effects of feeding rates in both anode and cathode on the sensor performance. Artificial wastewater (BOD_5_ of 102.4 mg/L) was fed into the anode; the current increased gradually from 3.7 mA to 5.2 mA with feeding rate increased from 0.48 to 1.07 mL/min (the cathodic flow rate was 5 mL/min). In cathodic chamber, oxygen was provided by feeding air-saturated tap water. The maximum current was 5.3, 5.7, and 5.9 mA when the cathodic flow rate was 5, 10, and 15 mL/min respectively (the anodic feeding rate was 1.37 mL/min), indicating the limitation of electron acceptor (i.e., oxygen in this case) at a high fuel feeding rate. Moreover, the effects of influent fuel types and coexisting ions on establishing reliable calibration curves between BOD concentration and output signals were also assessed [55]. Compared to methionine, phenylalanine and ethanol, monosaccharides was better fuel for electricity generation. This study also showed that Fe^2+^, Mn^2+^, Zn^2+^, and Cu^2+^ (below 5 mg/L) have an insignificant effect on the MFC performance, while Cr^6+^ (above 3 mg/L) reduced the current density by 5% to 7%.

### 2.2. Upper Limit of Measurement

**Figure 2:**
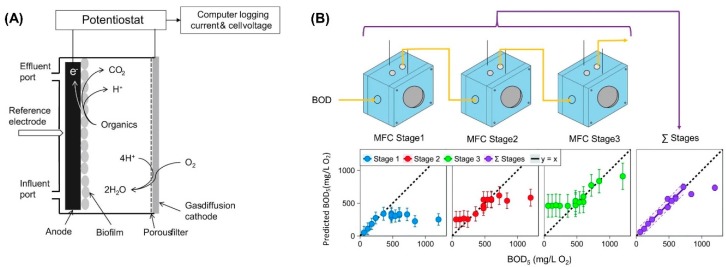
(**A**) Schematic diagram of the membrane-less single-chamber MFC-based BOD sensor [25]. (**B**) Schematic diagram of the three-stage MFCs as BOD sensor and compliance of predicted BOD_5_ values with five-day BOD test (BOD_5_). y = x is shown as the “ideal” prediction [57].

Not only the performance of MFC-based BOD sensors is affected by the operating parameters, but also the upper limit of the linear range is restricted by the capacity of the electrochemically active biofilm [56]. Various efforts have been made to increase the upper boundary. Modin et al. [25] constructed a special membrane-less single-chamber MFC (Figure 2A) for BOD monitoring. The applied voltage provided additional driving force for the electron transfer from microorganisms to anode, while the membrane-less configuration minimized the pH decrease in anolyte, which would disturb the biological activity on the anode. The retention time was also increased from 5 h to 20 h, which consequently improved the upper limit of linear range from 320 to 1280 mg/L. Another approach to improve the upper boundary was to use multi-stage MFCs connected in series [57]. The three-stage MFC array (Figure 2B) expanded the linear range (R^2^ = 0.97) to 720 mg/L at flow-rate of 0.52 mL/min by normalizing the total current output against BOD_5_ concentrations, while the single MFC configuration only had a linear range of 340 mg/L.

### 2.3. Oxygen Diffusion

The presence of other electron acceptors in the anodic chamber, such as nitrate and oxygen, was also found to reduce the current output of an MFC because they competed with anode for the electrons from biofilm [58]. Adding terminal oxidase and nitrate reductase inhibitors, e.g., azide and cyanide, could eliminate this effect [59]. However, they also caused “side effects” to the cellular metabolism [60,61], which could subsequently interrupt the extracellular electron transfer. Moreover, these chemicals are normally toxic to mammalian cells and the effluents need special treatment before being discharged into the environment. Using low gas-permeable membrane was also an efficient approach to eliminate the oxygen effect, since oxygen diffusion through the membrane was the major source of the oxygen present in the anodic chamber. Ayyaru et al. [24] fabricated a single-chamber MFC for BOD monitoring, using a sulfonated poly ether ether ketone membrane to prepare a membrane electrode assembly. The oxygen diffusion coefficient of the new membrane was only 10% of the Nafion membrane. Compared to Nafion-based system, the MFC with this new membrane showed improved current output by over 76% and the upper limit of the linear range increased by 62.5%.

### 2.4. Detection Limit

In addition to expanding the upper boundaries, improving the lower limits (i.e., the sensitivity) of BOD sensors is also required, because the surface water and secondary sewage usually contain low levers of biodegradable organic compounds [62]. Kang et al. [63] designed an oligotroph-type MFC-based BOD sensor which could detect as low as 5 mg/L BOD in the solution by reducing the O_2_ diffusion from the cathode to the anode with small membrane size. Besides, a platinum-coated graphite cathode with high catalytic activity toward oxygen reduction was used to facilitate the cathodic reaction. Alferov et al. [21] built an MFC based on immobilized *Gluconobacter oxydans* cells on a nitrate-pretreated graphite anode and 2,6-dichlorophenolindophenol was used to mediate the electron transfer. The lower limit of quantification for BOD measurement with this specific configuration was down to 0.34 mg/L. Immobilization of microorganisms on the anode reduced the internal resistance of the device while 2,6-dichlorophenolindophenol was able to shuttle the electron between *G. oxydans* bacteria and the electrode [64]. For smart biosensing, MFC-based biosensors were reported to be integrated with artificial neural networks [65], which could interpret a wide variety of electrical response peak and might be applied to detect low BOD values.

### 2.5. Response Time

The response time is a critical parameter to evaluate a BOD sensor. It is defined as the time required to reach a new steady-state after BOD variation [29]. Fuel feeding rate, external resistance, and cell structure were reported to affect the response time. Moon et al. [66] kept the fuel feeding rate of the MFC at 0.53 mL/min and obtained the shortest response time among all fuel feeding rates (0.35, 0.53, 0.65, and 1 mL/min). They could also shorten the response time from 2.1 to 1.4 h by lowering the external resistance from 100 to 10 Ω. Furthermore, scaling down the anode chamber from 25 mL to 5 mL was able to dramatically decrease the response time from 36 min to 5 min.

### 2.6. Cost-Effectiveness

Typically, membrane and cathode (catalyst) account for the majority investment required for an MFC reactor [67]. Intensive work, however, has been done to decrease their cost [68,69,70]. For example, two cheap membrane materials, i.e., a natural polymer (eggshell membrane) and a synthetic polymer (polydimethylsiloxane), were investigated by Chouler et al. [71]; comparable capabilities and sensitivities were obtained for the MFCs using these two membranes for BOD detections, compared to the one with an expensive Nafion membrane. In addition, Kharkwal et al. [70] utilized manganese dioxide (β-MnO_2_) rather than platinum as the catalyst for oxygen reduction in a single-chamber air-cathode MFC. It also gave identical results as the BOD_5_ values measured by conventional standard method, with slight variations in the range of 3%–12%. The system with β-MnO_2_ catalyst was operated for over one and half years and a linear relationship (R^2^ = 0.93) between voltage and BOD_5_ values (in the range of 33 to 160 mg/L) was still achieved, suggesting that the long stability of this catalyst was excellent. Furthermore, a membrane-less MFC with activated carbon as the cathode catalyst was examined [72]. Linear correlation (R^2^ = 0.99) was observed between the charge and the BOD concentration in a range from 80 to 1280 mg/L under the reaction time of 50 h.

## 3. MFC-Based Biosensors for Toxicity Detection

**Figure 3:**
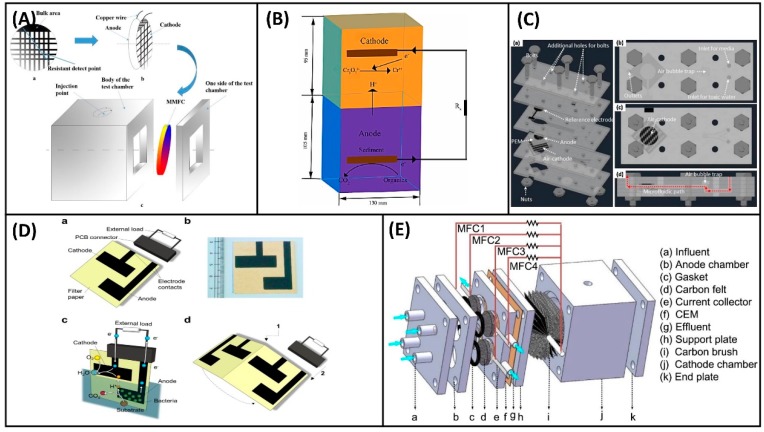
Photographs or schematics of MFC toxicity sensors: (**A**) membrane-based electrodes [77], (**B**) abiotic cathode sensing element [78], (**C**) miniature MFC with air bubble trap [79], (**D**) paper MFC [80], and (**E**) shared cathode [81].

The industrial revolution boosts the society development but also brings tons of different new-to-nature compounds into the water ecosystem [73]. Many of them are harmful to human as well as other living organisms and thus it is essential to detect and trace them. Traditional approaches include off-site chemical analysis using physicochemical methods, such as high performance liquid chromatography, gas chromatography-mass spectrometer, and liquid chromatograph-mass spectrometer [74]. These methods are normally time-consuming. However, real time detection is essential for sensor performance. MFC-based biosensors can be a good approach as it is directly based on the biotoxicity effects of the toxicants. The existence of toxic pollutants can inhibit the activity of electrogens and subsequently interrupt the current generated by MFCs [35]. The more toxic the substance is to the microorganism, the greater the current decreases. Therefore, different toxicity sensors can be built based on the relationship between the toxic substances and the amplitude of current reduction [75]. Toxicity sensors are mainly used to determine whether the concentration of toxic substances in an effluent exceeds the maximum concentration allowed by the regulations. Therefore, the concentration of MFC sensor for toxicity test is not aiming at the linear range as the case in BOD, but rather on the detection limit of the pollutants. So far, the detection limit of MFC-based toxicity sensors is still far away (tens to hundreds times higher) from the water quality standard published by the World Health Organization [76]. MFC-based toxicity biosensors could be generally grouped into four main categories based on the target pollutants: heavy metals biosensors, antibiotics biosensors, organic toxicants biosensors, and acidic toxicity biosensors. Some typical reactor configurations designed for specific toxicity detection are presented in Figure 3.

### 3.1. Heavy Metals

Heavy metals have relatively long half-life time (tens to hundreds of years) and can hardly be removed or reduced by microorganism. They would also accumulate in human body along the food chain and could cause health problems once reaching certain concentrations, despite some of them are essential for human health [82].

Heavy metal ions can inhibit respiration activities of microorganisms [83], which is the foundation for the current output of MFCs. Six heavy metal ions (2 mg/L, Cu^2+^, Hg^2+^, Zn^2+^, Cd^2+^, Pb^2+^ and Cr^3+^) were tested in a dual-chamber MFC system and their inhibition rate on current output was 12.56%, 13.99%, 8.81%, 9.29%, 5.59%, and 1.95%, respectively [84]. To improve the sensitivity and stability of MFC-based biosensors, Xu et al. [77] developed a flat membrane-based MFC sensor (Figure 3A) and tested it with two ions (Cr^6+^ and Ni^2+^). The voltage decreased to 40 mV from 180mV in 40 min after adding 10 mg/L Cr^6+^, and 50 mV in 6 min for 20 mg/L Cr^6+^. In contrast, injecting 20 mg/L Ni^2+^ into the anolyte only resulted in a slight voltage drop from 180 mV to 150 mV within 180 min. Faster voltage drop (45 min) was observed with higher concentration (50 mg/L Ni^2+^), but the change was in the same range.

Some MFCs were established for specific target compounds based on the principle that heavy metal ions can compete with the anode for electrons in the anodic chamber, which results in fewer electrons transferred to the cathode. Cr^6+^ can be reduced by Cr^6+^-reducing anaerobes under anaerobic conditions and serves as the terminal electron acceptor [85]. Thus, the cell voltage will be expected to decrease by increasing Cr^6+^ concentration when an MFC is developed using Cr^6+^-reducing anaerobes. As a proof of this concept, Cr^6+^-reducing bacteria, *Ochrobactrum anthropi* YC152, was inoculated in an MFC for the detection of Cr^6+^ [86]. The results demonstrated that the designed biosensor could quantitatively detect Cr^6+^ in the range of 0.0125–5 mg/L. A similar system was developed by Wu et al., using another Cr^6+^-reducing bacteria, *Exiguobacterium aestuarii* YC211, whose linear range was 2.5 to 60 mg/L [87].

In contrast to the negative effects, positive correlation between the ions and MFC outputs was also observed. A typical example was the ion-oxidizing bacteria. Ion(II) can be used as the sole electron donor in the anolyte by iron-oxidizing bacterial consortia. Tran et al. [88] inoculated this specific bacterial consortia in the anode to build an MFC-based sensor. A linear correlation between the current output and Fe^2+^ was obtained within the concentration of 3–20 mM.

Instead of sensing the heavy metal ions based on their biological effects on the electrogens, the sensor can also be built by applying them as cathode electron acceptors of the MFCs. The abiotic cathode sensing element was studied in MFC sensors to detect heavy metal ions (e.g., Cr^6+^, Cu^2+^) lately. Zhao et al. [78] utilized a sediment MFC (SMFC) to monitor Cr^6+^ in industrial wastewater (Figure 3B), in which Cr^6+^ was reduced at the cathode. The linear responsive range was between 0.2 and 0.7 mg/L, which well covered the maximum Cr^6+^ content (0.5 mg/L) allowed by the Chinese National Standard for Industrial Wastewater Discharges. The SMFC was also used to measure Cu^2+^, which acted as an electron acceptor and was eventually deposited on the cathode surface as Cu(0) [89]. A linear relationship (R^2^ = 0.87) was observed between the voltage increment and the Cu^2+^ concentration (5–160 mg/L).

Currently, MFC-based sensors were also applied to monitor heavy metal ions in tap water. An MFC sensor was built based on O_2_-reducing microbial cathodes for detection of toxic shocks in tap water [90]. Three heavy metal ions (Hg^2+^, Cr^6+^, Pb^2+^) were examined, and the detection limits for them were typically in the range of 1–10 mg/L.

### 3.2. Antibiotics

Antibiotics have saved millions of people, but the improper handle and discharge of antibiotics into environment have also interfered with the natural evolution process, causing many safety issues to microbial ecosystem and subsequently human beings [91]. Tracing and managing the discharge and distribution of antibiotics becomes an urgent issue concerning the future generations. Among all the antibiotics sensors, MFC is one of the real-time technologies to detect the antibiotics in field.

Wu et al. [92] constructed a single-chamber MFC with hydrophilic carbon cloth as the anode to detect tobramycin. At the concentration of 0.1, 0.24, and 0.47 g/L, no obvious effects could be detected. However, once the concentration reached 0.93 g/L or higher, a significant drop of the current output was observed. Less than half of the original current output could be retained after the addition of tobramycin. Interestingly, the current could be recovered after about hundreds of hours depending on the tobramycin concentration. This phenomenon demonstrated the robustness of the MFC-based sensors for tobramycin (maybe other antibiotics as well) measurement during long-term operation, because of the “self-healing” character of the electroactive biofilms in MFCs.

Schneider et al. [41] developed a fast approach for β-lactam antibiotics analysis by integrating miniaturized MFCs into a panel system. Two model microorganisms, *Escherichia coli* strain ATCC 25922 and *Staphylococcus aureus* strain ATCC 29213, were tested for the concept proof, and ten different β-lactam antibiotics (penicillin, ampicillin, cefoxitin, ticarcillin, cefazolin, cefuroxime, cefoperazone, cefepime, cefaclor, and imipenem) were examined at different concentrations ranging from 1 to 75 µg/mL. The antibiologic effects of these compounds, in terms of changes of the cell voltage output, could be measured in 2–4 h after injecting the cell suspensions into the MFCs, whereas the traditional Kirby-Bauer disc diffusion method for antibiotic test typically requires 24–48 h.

Another commonly used antibiotic, levofloxacin, was examined using a single-chamber MFC [38]. With sodium acetate as the carbon source in the anode, the MFC sensor could detect the levofloxacin up to 1000 μg/L. Linear relationship (R^2^ = 0.924) was obtained between the current output and the levofloxacin concentration in the range of 0.1–100 μg/L. Moreover, this MFC had operated for over 14 months while still generated steady electricity output, further demonstrating the advantages of MFC-based sensors for antibiotic detection in long-term operation.

### 3.3. Organic Toxicants

Organic toxicants, including organic nitrogen compounds, organic phosphate compounds, polycyclic aromatic hydrocarbons and polychlorinated biphenyl (PCBs), are commonly present in water, which may result in eutrophication and cause adverse effects on public safety [93,94,95].

Kim et al. [35] utilized a dual-chamber MFC to test the toxicity of diazinon (an organophosphorus compound) and PCBs, and the inhibition ratios resulting from diazinon and PCBs (1 mg/L) were 61% and 38%, respectively. Yang et al. [79] developed a single-chamber micro-sized MFC (Figure 3C) for monitoring of formaldehyde in water. This micro-sized system integrated a solid-state thin film Ag/AgCl reference electrode to poise an optimized anodic potential and a microscale air bubble trap to preclude microscale air bubbles from entering the MFC biosensor. When formaldehyde was introduced in the medium, the current decreased proportionally with its concentration ranging from 0.001% to 0.1%, while the anode potential was fixed at 0.2 V against the reference electrode. Recently, a single-component paper MFC (Figure 3D) was constructed and applied for chemical detections in water phase [80]. The biodegradable carbon-based electrodes were printed on a single sheet paper, and the anode was merged in the liquid phase while the cathode remained in the gas phase during the operation. The paper basement was functioned as (i) separator between electrode and (ii) also bridge for mass transfer because of the capillary force coming from the paper material. Formaldehyde was tested in the study; the addition of 0.1% (*v*/*v*) formaldehyde induced an immediate drop of the current output. Moreover, two MFCs could be printed on one paper and they were connected in parallel by being folded back-to-back. The stacked MFCs gave higher sensitivity to formaldehyde shock with the current output being completely diminished in 115 min, compared to 175 min for the single paper MFC.

In contrast to the inhibition effects, Chen et al. [40] established a dual-chamber MFC using p-nitrophenol (PNP) as a sole substrate. The reactor was inoculated with an aerobic strain, *Pseudomonas monteilii* LZU-3, and thus the anodic chamber was maintained in an aerobic condition (not the most common anaerobic condition in MFCs). Under the optimal operation parameters (the external resistance of 1000 Ω, the pH of 7.8 and the temperature of 30 °C), the cell voltage increased at higher PNP concentrations. A linear relationship (R^2^ = 0.98) was observed between the maximum voltages and the PNP concentrations in the range of 16 ± 5 to 44 ± 4.5 mg/L. While PNP was mixed with other aromatic compounds (5 mg/L of 2-nitrophenol, nitrobenzene and toluene), linear correlation between the cell voltage and the PNP concentrations (9 ± 4–36 ± 5 mg/L) could still be obtained.

### 3.4. Acidic Toxicity

The acidic toxicity is of prime interest to be monitored online because many types of toxic substances in wastewater, e.g., mine drainage, cause a sudden change of the pH [96]. A low pH value can inhibit the microbial activity and affect the growth of aquatic animals and plants, which accordingly reduces the self-purification ability of the water body and deteriorates the water quality.

Shen et al. [97] designed a single-chamber air cathode MFC and operated it in a continuous batch mode. The pH values of the influent (i.e., electrolyte in the working chamber) was modified by adding HCl. While the pH was maintained at three or four, the output voltage decreased rapidly and recovered overtime after stopping the addition of HCl. However, adjusting the influent’s pH to the value of two caused a catastrophe to the voltage output, which was probably caused by the irreversible damage of electrochemically active biofilm under strong acidic condition. Jiang et al. [81] constructed a cathode-shared MFC sensor array (Figure 3E) to detect acidic toxicity. This sensor array operated in continuous mode could guarantee the detection credibility because the cathode performance variation was avoided. After the MFC array reached a steady state, an acidic toxicity shock was applied using acidified anolyte. When the pH reduced from six to four, the voltage decreased from 200 mV to 0 mV immediately. The threshold pH value might vary with different biofilm composition; nevertheless, this phenomenon enables a potential approach to access the pH in the water based on the interruption of cell voltage of MFCs.

In addition to monitoring acidic toxicity in water, acid rain damage was also reported to be detected. In plant MFCs (PMFCs), rhizosphere microbes can generate electrical current by degrading the organic excretes of the rhizodeposits [98], so any changes of the bioavailable substrates concentration can affect the electrical current. Li et al. [99] constructed PMFCs to assess acid rain damage, using mixed solution of concentrated H_2_SO_4_ and HNO_3_ to simulate acid rain. Artificial acid rain could hurt the leaves of rice plant and thus reduced photosynthesis activity that correlates with rhizospheric electrochemical activity. Immediate and repeatable current drops were obtained within 2 min after simulated acid rain was sprayed on plant leaves, which were in good correspondence to the changes in rhizospheric organic concentration.

## 4. Other Applications

### 4.1. DO Detection

Molecular oxygen dissolved in water (DO) is an essential indicator and common parameter in water management [100]. For example, its changes were shown to reflect the organic pollutants contents flowing into a freshwater lake [101]. It also provides important information about biological and biochemical reactions in the aquatic environment, while the DO level is a naturally selective pressure for different lifestyles of microorganism. The accurate measurement of DO in the field using Clark-type oxygen sensors is challenging, because they are significantly affected by the environmental parameters such as pressure [100]. An alternative strategy for DO measurement is with MFCs. Compared to the Clark-type oxygen electrodes, MFC-based biosensors are more stable against the environmental conditions and can provide real-time monitoring in the field. The fundamental principle of MFC-based DO measurement is based on the cathode behavior. The cathode efficiency is a constraint for the MFC performance [102] while oxygen, as the final electron acceptor, can largely determine the cathodic reduction rate and thus the current output. A Monod-like kinetic correlation between the current density and DO values was found by Oh et al. [103], with the half saturated DO value of 1.74 mg/L.

Measuring DO in real time can also be important for studying the aquatic ecosystem. Recent years, periodic oxygen stratification was found in some shallow-water freshwater high-nutrient lakes, which eventually leads to the formation of “dead zone” in the lakes [104,105]. Monitoring DO concentration can serve as an early warning signal for the risk of a “dead zone” in a lake. For this purpose, Song et al. designed a multi-cathode sediment MFC system that includes multiple cathodes placed at different depth of water for in situ, continuous and on-line monitoring of dissolved oxygen (DO) concentrations along lake depth [33,106]. Linear correlation (R^2^ = 0.9576) was observed between voltage and DO in the range of 0–9 mg/L.

### 4.2. Microbial Activities Detection

Traditional microbiology approaches for microorganism screening and phenotyping are quantitative but time-consuming and labor-intensive. In this case, MFC was proposed to be a fast and easy method to collect the first-hand information of the microorganism and its general lifestyle [107]. The mechanism is basically according to the microbial metabolic activities (thus electron output to anode) specifically determined by the given environmental conditions. One example was reported by Miller et al. to predict the presence of arsenate-respiring bacteria in soda lakes because of the competence of arsenate against anode for electrons [108]. Another hypothetic application was proposed by Abrevaya et al. [109] to use MFC to detect living (micro)organisms in other planets assuming they could also export electrons during their life process. In more practical aspect, MFC has been shown to be a good approach for bioprocess monitoring. Liu et al. [110] designed a flow-cell MFC to trace the anaerobic digestor performance, and found the current output changes correlated to variations of the operating parameters, e.g., pH, gas flow rate and also COD for up to 6 months. Moreover, Sun et al. [111] also reported the current density of MFC was linear toward acetate concentration up to 20 mM, and only minor interference could be detected from the other volatile fatty acids existed in the anaerobic digestor. These results proposed the possibility to develop an MFC-based senor to monitor the metabolic turnover rates of organic compounds in the anaerobic digestor.

### 4.3. Other Parameters

Accurately and rapidly detecting hydrogen peroxide (H_2_O_2_) is of great importance in industrial, environmental and physiological applications. The detection of H_2_O_2_ by MFC-based sensors was first reported by Liu et al. [112]. Graphite cathode and bioanode were used as the H_2_O_2_ sensing element and the power supplier, respectively; AEM and CEM were applied to establish two types of H_2_O_2_ sensors. The sensors displayed an exponential fitting relationship between the current response and the natural logarithm of H_2_O_2_ concentration ranging from 1 to 2000 mM. Although most studies focus on water quality monitoring, MFC-based sensors were also applied to detect air quality. Carbon monoxide (CO), one of the most hazardous gases for human health, was detected by MFC-based sensors based on the principle that CO inhibits bacterial activity in the anode and thus reduces electricity production [113]. The voltage drop was proportional to the CO concentrations in the range of 10–70% (*v*/*v*), and the response time of the biosensor was 50–60 min. Instead of using a bioanode sensing element, Jiang et al. constructed a gas diffusion–biocathode sensing element to detect formaldehyde, which could be applied for monitoring gaseous pollutants [114]. In addition, Zhang et al. [115] proved the positive correlation between the voltage signals of MFC-based sensors and methane emission flux in paddy fields of China, developing a new method to evaluate methane emissions.

### 4.4. Powering External Sensors

MFC can also integrate with other units to expand its applications and overcome the limitation of low selectivity, which has been a main limiting factor of MFC-based biosensors. One example is to use MFC as an in situ power source for other commercial sensors. Zheng et al. [116] applied MFC to power a wireless sensor for temperature and humidity detection. To achieve this, a power management unit including capacitor and convertor was introduced to raise the voltage output of MFC to meet the requirement of the sensor. Similar approach was also confirmed by Khaled et al. [117]. The MFC, connected to a power management unit, could be used power different sensors for tracing such as CO_2_, humidity, temperature, pH, and even water level. Compared to other battery-based power source, MFC requires much less maintenance and is superior in long term operation especially for remote areas.

## 5. Challenges and Perspective

As an analytical technology, MFC-based biosensor has gained a growing interest word wide because it is self-powered device using whole-cells. As described above, significant progress has been made in the field, especially in BOD and toxicity detection. However, there are still some problems needed to be addressed before it becomes a mature sensing technology accepted by scientific communities.

First, more investigations on the stability of MFC-based biosensors are urgent, which are often overlooked. MFC use bacteria as the catalyst, which are self-renewable. However, bacteria can evolve rapidly in response to environmental changes during long term operation. As a result, the sensitivity, selectivity, and reproducibility of the biosensors will be adversely affected.

Besides, screening of bacteria having high extracellular electron transfer rate might be a good strategy to improve the MFC-based biosensors. Construction of genetically engineered microorganism can also be an alternative to enhance the biosensor performance. Identification of electrogenic gene associated with electron transfer and metabolic pathway not only enhances the existing applications of MFC-based biosensors, but also expands their use for other analysis.

Further, the detection limit of MFC-based biosensors, especially for toxicity detection, is typically significantly poorer than the water quality standard of the World Health Organization. Therefore, the detection limit should be greatly lowered in order to meet the requirement.

Finally, the water quality has great influence on the electrical signal output of the MFC-based biosensors and therefore signal interference might occur, especially in complex aquatic environments. For example, a BOD variation can weaken the signal for toxicants. Jiang et al. [118] systematically studied the influence of the background organic matter concentration on the performance of MFC-based toxicity sensors. To avoid the signal interference in the combined shock of BOD and toxicity, they operated two MFC sensors with high and low organic matter concentrations and compared the signal output with the pre-made response chart to make qualitative distinctions. Jiang et al. [42] also applied biocathode for toxicity monitoring in order to avoid the combined shock of BOD and toxicity. Most MFC-based toxicity sensors could only assess the overall toxicity while only a few studies evaluated the toxicity of a specific agent. Specific pure cultured or genetically engineered microorganism can be applied in MFC-based biosensors for a specific monitoring, such as *Pseudomonas monteilii* LZU-3 used for the detection of PNP [40]. A multi-chamber MFC sensor with each chamber inoculated with different electrogenic microorganisms was also developed to monitor different target toxic agents in a same stream [119]. However, it is still a big challenge to improve the specificity of MFC-based biosensors. Accordingly, more studies are necessary to apply molecular biology or other modern techniques to obtain higher specificity.

## 6. Conclusions

MFC as an analytical tool has been developing very fast over the past two decades. Its application has been expanded from BOD measurement to toxicity detection, DO detection, microbial activity analysis or as a power source for other sensors. It exhibits unique advantages in these applications, such as easy construction, simple operation, low cost and in situ monitoring. Some of the MFC-based biosensors are commercially available. With advances in materials and microbiology, especially electrogenic bacteria, MFC-based biosensors may eventually become approved standard methods.

## Figures and Tables

**Table 1:** Summary of analytical performance of MFC-based biosensors.

Parameter	Anode	Cathode	Separator	Detection Range	Response Time	Ref.
BOD	AG	AG	PEM	0.34–9.6 mg/L	30–130 min	[21]
	GF	CC/Pt	–	5–120 mg/L	132 min	[22]
	GF	GF/Pt	CEM	20–200 mg/L	5–36 min	[23]
	CC	CC/Pt	SPEEK	0–650 mg/L	80 min	[24]
	GR	CP	PF	32–1280 mg/L	300–1200 min	[25]
COD	CF	AC	Ceramic	57.7–149.7 mg/L	3 min	[26]
	CC	CC	PEM	3–164 mg/L	2.8 min	[27] rmatting Citation}
	GG	AC	–	0–500 mg/L	N/A	[28]
	CC	CP/Pt	CEM	100–500 mg/L	31–825 min	[29]
VFAs	CR	CR	–	0.5–2 mM	N/A	[30]
	CB	TWWM/Pt	AEM	5–100 mM	60–240 min	[31]
DO	CP	CP	PEM	0–8.8 mg/L	<4 min	[32]
	GF	GF	–	0–9 mg/L	N/A	[33]
Ni^2+^	GP	GP	CEM	10 mg/L	30 min	[34]
Pb^2+^	GF	GF	CEM	1–5 mg/L	20–120 min	[35]
Hg^2+^	GF	GF	CEM	1–5 mg/L	20–120 min	[35]
Cr^6+^	CC	CC/Pt	–	1–8 mg/L	74 min	[36]
Cu^2+^	CC	CC/Pt	CEM	5–7 mg/L	240 min	[37]
Cd^2+^	CC	CC	CEM	0.1–100 μg/L	12 min	[27]
Levofloxacin	SCE	CC	–	0.1–1000 μg/L	10 min	[38]
SDS	GP	GP	PEM	10–50 mg/L	N/A	[39]
p-Nitrophenol	CF	CF	PEM	10–50 mg/L	27 min	[40]
β-lactam antibiotics	ENIG	Graphite	PEM	1–75 µg/mL	120–240 min	[41]
Formaldehyde	GF	CF	CEM	0.0005–0.01%	10–240 min	[42]
microbial activity	CP	CP/Pt	PEM	0–13 nmol/L	<186 min	[43]

BOD, biochemical oxygen demand; COD, chemical oxygen demand; VFA, volatile fatty acids; DO, dissolved oxygen; CB, carbon brush; CC, carbon cloth; CF, carbon fiber; CP, carbon paper; CR, carbon rod; GF, graphite felt; GG, graphite gravel; GP, graphite plate; GR, graphite rod; AC, activated carbon; AG, activated graphite; SCE, saturated calomel electrode; Pt, platinum; TWWM, titanium woven wire mesh; ENIG, electro less nickel immersion gold; AEM, anion exchange membrane; CEM, cation exchange membrane; PEM, proton exchange membrane; PF, porous filter; SPEEK, sulfonated poly ether ether ketone; SDS, sodium dodecyl sulphate; N/A, not available.
